# Potential Role of Cannabidiol on Sports Recovery: A Narrative Review

**DOI:** 10.3389/fphys.2021.722550

**Published:** 2021-08-03

**Authors:** Daniel Rojas-Valverde

**Affiliations:** ^1^Clínica de Lesiones Deportivas (Rehabilitation and Readaptation), Escuela Ciencias del Movimiento Humano y Calidad de Vida (CIEMHCAVI), Universidad Nacional, Heredia, Costa Rica; ^2^Centro de Investigación y Diagnóstico en Salud y Deporte (CIDISAD), Escuela Ciencias del Movimiento Humano y Calidad de Vida (CIEMHCAVI), Universidad Nacional, Heredia, Costa Rica; ^3^Núcleo de Estudios para el Alto Rendimiento y la Salud (NARS), Escuela Ciencias del Movimiento Humano y Calidad de Vida (CIEMHCAVI), Universidad Nacional, Heredia, Costa Rica

**Keywords:** sport, fatigue, CBD, injury, sleep, exercise

## Abstract

The use of cannabidiol (CBD) among athletes is becoming extensive and frequent. This could be due to the elimination of CBD from the list of prohibited substances by federations and international institutions of sport. The legalization and resulting production, and commercialization of CBD, could increase its intake in sports professionals. This commercialization of cannabinoids has fueled a race to study their properties, benefits, and risks for health and performance in athletes. Although there is evidence that suggests some beneficial properties such as anxiolytics, antidepressants, anti-inflammatory, and antioxidants among others, the evidence presented so far is neither clear nor conclusive. There are significant gaps in knowledge of the physiological pathways that explain the role of CBD in sports performance. This mini-review examines evidence suggesting that CBD has the potential to be used as a part of the strategies to recover from fatigue and muscle damage related to physical and cognitive exertion in sports.

## Introduction

Recovery has become a crucial topic in recent sports research and could determine physical (Trecroci et al., 2020b), physiological (Rojas-Valverde et al., 2018), and cognitive (Trecroci et al., 2020a) performance, considering the high frequency and density of competitions. This has led the researchers, coaches, and athletes making plans and managing recovery strategies as part of the general exercise prescription (Martínez-Guardado et al., 2020). The physical, physiological, and cognitive effort usually provoke a cascade of structural and functional adjustments that need to be identified, monitored, and controlled to optimally recover the functional capacities of the athlete (Ament and Verkerke, 2009). Commonly, central and peripheral fatigue related to physical exercise manifests itself as pain, weakness, inflammation, loss of functional mobility, decreased force generation, feeling of tiredness, alteration of vital signs, and reduced concentration, among others.

Over the last few years, many methods and means of recovery from fatigue have been tested (Rawson et al., 2018). One of the best known strategies is the intake of plant-derived products such as ginseng (Rojas-Valverde et al., 2020), green tea (Machado et al., 2018), cherries (Bell et al., 2014), curcumin (Fernández-Lázaro et al., 2020), spinach (Bohlooli et al., 2014), and beetroot (Rojas-Valverde et al., 2020). These organic products have shown anti-inflammatory, antioxidative, and analgesic properties as other cognitive benefits that promote recovery from exercise-related fatigue (Bongiovanni et al., 2020).

Recently, the World Anti-Doping Agency has removed some products from their list of prohibited substances for athletes. This is the case of cannabidiol (CBD), a phytocannabinoid clustered among the cannabinoids extracted from the *Cannabis sativa* plant (Campos et al., 2012). Unlike tetrahydrocannabinol (THC), CBD does not cause psychotomimetic and psychotropic reactions (WHO, 2017) for which there is no evidence of dependence or abuse, but causes mild and infrequent side effects (Stout and Cimino, 2014). On the contrary, CBD use is not only extensive among athletes (Docter et al., 2020), but it has been shown to have specific properties that help to treat chronic pain, spasticity, mood and sleep disorders, immunodepression, inflammation, oxidant effects, and anxiety in clinical patients (McPartland et al., 2015; Whiting et al., 2015; Nichols and Kaplan, 2019; Pinto et al., 2020). These effects could improve and accelerate recovery caused by a prolog or intense physical, physiological, and cognitive efforts as in sports (Higgins et al., 2017).

Considering that CBD has gained wide acceptance for medicinal and recreational use, its use among athletes is imminent (Docter et al., 2020) even though its the physiological, physical, and cognitive effects are not fully understood (Nichols and Kaplan, 2019), and it seems premature to make specific recommendations and to award all the above mentioned benefits (Gamelin et al., 2020). Consequently and considering the need to clarify these issues, this narrative review aims to present the scientific evidence around the potential benefits of CBD as an ergogenic aid to promote a better and faster recovery between efforts related to physical exercise and sport. Given the absence of evidence directly exploring the CBD potential in sports recovery, this review synthesizes the preclinical and clinical findings that support its use and testing in future research protocols. This narrative review was performed considering the scale for assessment of narrative review articles (Baethge et al., 2019).

## Prevalence in the Use of CBD Among Athletes

With the exclusion of CBD from the prohibited substances in 2018, and even before, the use of CBD among athletes has considerably increased and is still accelerating (Leas et al., 2019). Cannabinoids are considered the second most commonly used substance among contact sports athletes replacing nicotine (McDuff et al., 2019). Evidence has shown that a third of cyclists, triathletes, and runners are or have been cannabinoids users (mostly ≥ 40 years of age, male, THC + CBD consumers ≤3 times weekly, and exercise 5–7 days per week) (Zeiger et al., 2019). Also, a quarter of university athletes report using cannabis-related products (Docter et al., 2020). Especially in contact sports like rugby, the use rate of CBD is 28%, increasing with age, and reporting pain relief and sleep quality improvements as perceived benefits (Kasper et al., 2020).

Despite the extensive use of CBD and the fact that international sports organizations have now allowed for it to be used, some CBD products have been shown to contain significant levels of other banned cannabinoids, like THC (Lachenmeier and Diel, 2019). Besides, there is evidence of the use of synthetic cannabinoids, such as JWH-018 and JWH-073, with limited regulation (Heltsley et al., 2012). Athletes require more information and advice, as product labels can be misleading about whether they contain THC, meaning there are risks in terms of violating anti-doping rules (Mareck et al., 2021).

## Physiological Mechanism Framing CBD

The effects of CBD on physiological and cognitive functions are mediated by the endocannabinoid system, which has regulatory functions to maintain homeostasis (VanDolah et al., 2019). During exercise, the cannabinoid system mediates some central and peripheral effects of exercise as bliss, peacefulness, and euphoria (Carek et al., 2011). Endocannabinoids [e.g., anandamide and 2-arachidonyol (2AG)] as cannabinoids activate the type-1 (CB_1_) and type-2 (CB_2_) cannabinoid receptors, such as *N*-acylethanolamines (De Petrocellis and Di Marzo, 2009), leading to appetite-suppression, anti-inflammatory, anxiolytic, and antiproliferative effects as exercise do. CBD inhibits the degradation and uptake of endocannabinoids as anandamide, leading to an increase in endocannabinoid–receptor binding. CB_1_ and CB_2_ are present mostly in the central nervous and peripheric nervous system, respectively.

Also, cannabinoids and endocannabinoids are involved in brain-derived neurotrophic factor release (e.g., neurogenesis and neuronal plasticity), glucocorticoids release (e.g., mood control by suppressing depression and anxiety), dopamine release (leading to rewarding), and fatty acid amide hydrolase release (e.g., analgesic effects). All these responses overlap with the positive benefits of exercise (Tantimonaco et al., 2014). These effects are provoked by stimuli of TRPV1 ions canals (Vanilloid receptors) leading to antinociceptive effects, stimuli of CB_1_ and CB_2_ receptors causing relaxing effects *via* neurodepression and inhibition of cytokines release, respectively, and activation of 5HT_1__A_ receptors promoting serotonin caption in the postsynaptic neuron causing mood state regulation.

## Inflammation and Proliferation

Inflammation and oxidative stress underlie many human chronic and acute health conditions and pathologies. In this sense, and considering that exercise-related damage and fatigue mediate inflammation, proliferation, and oxidative stress in most cases, it is hypothesized that CBD-related inhibitions in oxidative stress and neuroinflammation could have some therapeutic potential in sports research (Gamelin et al., 2020). This statement is based on evidence suggesting that CBD could induce changes in cortisol release, regulating inflammatory response to injury (Zuardi A. et al., 1993; Yeager et al., 2010). This mediation is due to the interaction between CBD CB_1_, and CB_2_ cannabinoids and adenosine receptors, leading to reduced cytokine levels and downregulating overreactive immune cells (Booz, 2011; Hill et al., 2012; Burstein, 2015). Also, CBD intake seems to mediate processes associated with gastrointestinal damage protection, due to inflammation, and promote healing of skeletal injuries (McCartney et al., 2020).

During exercise, mainly those actions with a high component of eccentric contraction are potentially and particularly damaging to the sarcolemma. This damage is fetterless in response to a disruption of the permeability of muscle cell membrane and basal lamina, allowing Ca^2^^+^ to reduce fiber electrochemical gradient. If the damage in the sarcolemma is relatively low, ATPase pumps attract Ca^2+^ and the damage is still reversible. Besides, if there is a Ca^2+^ overload, a degradation of the structural and contractile proteins could be provoked. The subsequent event is called the inflammatory cascade, recognized by the activation of macrophages and other phagocytic cells during the first 2–6 h after injury and prolonged for days (Armstrong et al., 1991; Burstein, 2015).

Additionally, CBD (300 mg) has been shown to induce changes in glucocorticoids as cortisol in humans (Zuardi A. W. et al., 1993), one of the primary homeostatic regulators of the inflammatory response to injury (Yeager et al., 2010). This is supported by a recent narrative review in sports, suggesting the potential anti-inflammatory effect in humans and the possible role in the performance of the athletes (McCartney et al., 2020). This affirmation is theoretically based on the suggested CBD capacity to interact with receptors involved in controlling inflammation as CB1 cannabinoid, CB2 cannabinoid, adenosine A2A, and also in reducing the levels of some cytokines, such as interleukin-1 (IL-1) and tumor necrosis factor α (TNFα), and downregulating overreactive immune cells reducing the impact of collateral inflammatory damage of tissues (Booz, 2011; Hill et al., 2012; Burstein, 2015). There is also evidence suggesting the CBD potential to promote the release of arachidonic acid, leading to greater healing capacity as a result of core regulation of growth signals mediated by proresolving substances, such as lipoxin A4 and 15d-PGJ2 (Burstein, 2015).

It is also known that the interplay between inflammation and oxidative stress underlies many human diseases due to tissue and organ damage. In this regard, in sports, it is hypothesized that CBD-related inhibitions in oxidative stress and neuroinflammation could have some therapeutic potential in sports research (Gamelin et al., 2020).

## Pain and Soreness

Cannabidiol has been commonly used for its analgesic properties (Kogan and Mechoulam, 2007) in a variety of pain disorders (Starowicz and Finn, 2017). CBD consumption could exhibit a beneficial effect over edema and hyperalgesia (Burstein, 2015; Hill et al., 2017), acting directly on the central nervous system and leading to sedative effects (Zuardi A. W. et al., 1993). The idea of considering CBD as an antinociceptive agent is based on the efficiency of treating the pain associated with proinflammatory cytokine release due to the activation of Vanilloid receptors, provoking antinociceptive effects and reducing the perception of pain (Booz, 2011). CBD could inhibit presynaptic neurotransmitters and neuropeptide release, modulate postsynaptic neuronal excitability, activate the descending inhibitory pain pathway, and reduce neuroinflammatory signaling (Starowicz and Finn, 2017).

Cannabidiol (300–400 mg) intake seems to have sedative effects on humans apparently acting directly on the central nervous system (Zuardi A. W. et al., 1993), supported by the idea that CBD exhibited a beneficial action over edema and hyperalgesia (Burstein, 2015; Hill et al., 2017). In this regard, drugs and substances such as Sativex, THC, and CBD are approved for the treatment of both central and peripheral neuropathic pain. This pain syndrome is associated with microglia activation and subsequent cascade of proinflammatory cytokines such as IL-6, IL-1β, and TNF (Booz, 2011). This evidence supports the idea of CBD use as an antinociceptive agent. Together with a neuroprotective quality, this effect was also found in a recent systematic review on the outcome of CBD intake in relation to its potential use as a sport-enhancing performance substance (McCartney et al., 2020). It still is unclear how CBD acts in relation to the pain cascade and pathways (Anthony et al., 2020). CBD has shown its potential to treat and manage pain in diseases and pain disorders, and based on this evidence CBD seems to have a potential effect on treating swelling and preventing soreness after strenuous exercise, but more evidence is required to make a clear statement.

## Sleep Disorders

Overreaching and overtraining are often presented in athletes due to high training loads accompanied by subsequent insufficient recovery between efforts (Fox et al., 2020). These abovementioned states are usually accompanied by sleep disorders and higher sleep disturbance, leading to poor sleep quality (Hainline et al., 2017). CBD consumption could stimulate the endocannabinoid system modulating sleep disorders and the sleep–wake cycle (Murillo-Rodríguez et al., 2020). Promising, but no specific, evidence suggests using cannabinoids like CBD to reduce sleep disorders in athletes or even in healthy or pathologic humans. Endocannabinoid system receptors as anandamide and type-1 are associated with sleep-promoting effects, but the physiological mechanism is not fully understood and is based mainly on preclinical studies (Suraev et al., 2020).

## Cognition and Mood

Evidence has shown that acute and single administration of CBD could have anxiolytic (Zuardi A. et al., 1993) and antidepressive effects through the activation of 5-HT1A receptors (Booz, 2011). Although the reported results are promising for sports recovery, evidence suggests no long-term impact on cognition or mood state due to prolonged use of CBD (Allendorfer et al., 2019; Martin et al., 2019). Also, the link between CBD consumption and the possible effect on exercise-related recovery is primarily clinical and preclinical studies, mostly in participants with background pathology (McCartney et al., 2020). In this sense, more in-depth analysis is needed in the population of athletes to reach a conclusive statement.

## Future Research and Limitations

As interest in the use of CBD in athlete recovery continues to grow, more research is required to better understand the physiological mechanism. The potential benefits, efficacy, and purported safety profile when consuming CBD prior to, during, and after training or competition should be explored. Future research in the field of sports science and medicine must focus on understanding the role of CBD in physiological mechanisms such as inflammatory cascade, neuroprotection, analgesic and anxiolytic pathways, muscle enhancement, and neuromechanical function.

New randomized placebo-controlled studies should consider the different etiologies of fatigue and damage, individualities and disciplines, and special needs and characteristics. Other potential research areas are, but are not limited to, optimal dosing depending on physical and physiological load; effectiveness regarding administration timing; chronic and acute effects; cumulative responses with other recovery strategies; differences in tolerance and effectiveness by sex, professional level, and fitness level; and other individual conditions and situational factors. Besides, more information is needed around the understanding of CBD inflammatory signaling as an essential factor in the recovery process. The effectiveness of CBD vs. conventional medications should be assessed.

This narrative review must be analyzed in light of some limitations. Though the main evidence about the use of CBD in sports was reviewed, this systematic review lacks explicit criteria for article selection and inclusion. In this sense, a systematic review could strengthen the actual conclusions and better present the preclinical and clinical evidence supporting the use of CBD in sports recovery. In this sense, a systematic review could better present settings of tests, study designs, demographics of participants, and main conclusions of the recent evidence.

## Conclusion

Evidence supporting the potential use of CBD as an ergogenic aid to improve the efficacy and efficiency of recovery processes during exercise and sport-related fatigue seems promising. Still, there is not enough information to be conclusive. CBD appears to have some properties that could boost exercise recovery as an anti-inflammatory, neuroprotective, analgesic, anxiolytic, and pain reliever. Still, due to the lack of studies in elite athletes, there is a need for a better understanding of the effects of CBD as a physiological, physical, and cognitive recovery agent.

More evidence and higher-quality studies are required in populations related to sports science and exercise medicine to be able to give recommendations regarding the dose and frequency of consumption as well as the specific prescribing of CBD according to the intensity and duration of the effort, as well as the role of essential characteristics such as body composition, general health, and other situational factors in its effect. Also, considering the lack of regulations in CBD production and indiscriminate consumption, athletes must be cautioned due to the high risk of testing positive in the doping tests.

Cannabidiol seems to have anti-inflammatory, neuroprotective, analgesic, anxiolytic, and pain-relieving properties which can be potential mediators of recovery in athletes during regular training and competition. To confirm these effects, more scientific evidence in specific sport-related populations is necessary. There is a need for confirmatory analyses using randomized, placebo-controlled trials testing acute, and chronic effects of different dosing prescriptions. This study must consider some fundamental particularities of sports as a great variety of biological and situational conditions that promote fatigue, the characteristics of each discipline during training and competition, as well as the individual peculiarities of athletes, their tolerance and response to CBD intake, and the combined effect of CBD administration with other physical and nutritional aids.

Since training and competition leads to a structural and functional imbalance due to strenuous effort, CBD intake could potentially promote restoration of physical performance. The CBD physiological mechanisms of action, mixed with other recovery protocols, could help to reduce the accumulated fatigue evident over a tournament of consecutive efforts. The above may depend on pointing to multiple mechanisms to provoke global functional recovery in sports. Much evidence is needed to support this conclusion, but the proposed evidence looks promising.

Considering the relatively common use of both cannabis and CBD alone among athletes, there is a clear need to improve scientific understanding of the effects of CBD use on the fatigue, damage-related recovery, and performance of athletes. Greater scientific progress is needed, mostly on the execution of experimental trials, allowing a greater understanding of both critical positive and negative outcomes for the final benefit of the athletes in exercise-related recovery and performance. Also, the evidence resulted could give new clinical guidance to prescribe CBD during the recovery process of an athlete and other possible applications. The potential therapeutic benefits of CBD administration have been downplayed for years but, the actual scenario could facilitate the boost of the knowledge around this natural compound and its effects. Besides, from an administrative point of view, clearer and overarching policy for the use of cannabis in sports need to be considered and adopted.

Finally, athletes have to create an optimal internal environment to increase the function of endocannabinoids. In this sense, besides regular exercise, athletes must control weight, manage stress and competition-related anxiety, and minimize environmental exposure to contaminants and other toxic substances. These cannabimimetic practices would create the ideal environment for improving the endocannabinoid action in recovery.

## Author Contributions

DR-V carried out the original idea conceptualization, literature search and systematization, writing the original draft, critically revising the manuscript, funding, and approving the final manuscript.

## Conflict of Interest

The author declares that the research was conducted in the absence of any commercial or financial relationships that could be construed as a potential conflict of interest.

## Publisher’s Note

All claims expressed in this article are solely those of the authors and do not necessarily represent those of their affiliated organizations, or those of the publisher, the editors and the reviewers. Any product that may be evaluated in this article, or claim that may be made by its manufacturer, is not guaranteed or endorsed by the publisher.
